# Gender inequalities in mortality in Lithuania: the HAPIEE Study

**DOI:** 10.1093/eurpub/ckac131.118

**Published:** 2022-10-25

**Authors:** H Pikhart, A Tamosiunas, J Pikhartova, E Paulikaite

**Affiliations:** 1 RECETOX, Masaryk University, Brno, Czechia; 2 Institute of Epidemiology and Health Care, UCL, London, UK; 3 Institute of Cardiology, Lithuanian University of Health Sciences, Kaunas, Lithuania; 4 Vilnius University, Vilnius, Lithuania

## Abstract

**Introduction:**

Gender inequalities in morbidity and mortality are important problem in countries of Central and Eastern Europe. Mortality difference between men and women in Lithuania is one of the largest in Europe. The aim of this analysis was to identify factors that influence this difference in population-based sample of middle- and older-age men and women from Lithuanian arm of the HAPIEE Study.

**Methods:**

Data come from the Lithuanian part of international Health, Alcohol and Psychosocial Factors In Eastern Europe (HAPIEE) longitudinal cohort study. The analytical sample included 3729 women and 3062 men aged 45-74 years at the study baseline. The study outcomes were all-cause, CVD and cancer mortality (mean follow-up approx. 10 years). Gender difference in study outcomes, and the role of wide range of socioeconomic, demographic, behavioural, metabolic and psychosocial covariates in this association was tested by regression modelling.

**Results:**

There were 913 deaths (576 in men) during the follow-up. Men had higher odds of mortality compared to women: for all-causes OR 2.42 (95% CI 2.09-2.81); for CVD 2.48 (2.03-3.08); for cancer 1.92 (1.54-2.38). BMI was identified as an effect modifier for all-cause and cancer mortality. When adjusted for confounders such as smoking, alcohol consumption or paid work, and stratified for BMI, the gender difference reduced a little for CVD mortality but remained virtually unchanged for all-cause and cancer mortality.

**Conclusions:**

We found substantial and statistically significant gender inequalities in mortality in this Lithuanian study. We found that men aged 45+ years were approximately two times more likely to die than women, with CVD mortality difference being even larger. Additionally, most of gender difference in mortality remained unexplained by the main social, psychosocial, behavioural and metabolic risk factors.

**Key messages:**

• Large gender inequalities in mortality have been observed in this Lithuanian study of middle- and older-age men and women.

• Most of the inequalities have not been explained by available social, psychosocial, behavioural and metabolic risk factors.

